# Barrier effect and wound healing activity of the medical device REF-FTP78 in the treatment of gastroesophageal reflux disease

**DOI:** 10.1038/s41598-022-10171-6

**Published:** 2022-04-12

**Authors:** Tiziana M. G. Pecora, Ortensia Ilaria Parisi, Walter Bertin, Barbara Ragazzo, Marco Dattilo, Norma Scigliano, Rocco Malivindi, Fabio Amone, Francesco Puoci

**Affiliations:** 1 Labomar SpA, via Nazario Sauro 35/I, Istrana, 31036 Treviso, Italy; 2 Department of Pharmacy, Health and Nutritional Sciences, University of Calabria, 87036 Rende, CS Italy; 3 Macrofarm S.R.L., c/o Department of Pharmacy, Health and Nutritional Sciences, University of Calabria, 87036 Rende, CS Italy

**Keywords:** Diseases, Gastroenterology, Health care

## Abstract

REF-FTP78 is a class IIb medical device present on the market with different trade names and developed for the treatment of gastroesophageal reflux disease (GERD). This medical device is based on polysaccharides from *Aloe Barbadensis* and fucoidans from brown seaweeds, such as *Undaria pinnatifida* and *Fucus vesiculosus*, and aims to exert a protective effect on the esophageal mucosa against the noxious components of refluxate. The present study reports on the efficacy of REF-FTP78 devoting a particular attention to the barrier effect and wound healing properties, combined with antioxidant and anti-inflammatory activities. Film-forming properties and barrier effect were investigated on in vitro reconstructed human esophageal epithelium, through TEER measurement and evaluation of caffeine and Lucifer yellow permeability, and in an ex vivo swine model of esophageal mucosa damage. Antioxidant and anti-inflammatory properties were evaluated in terms of scavenging activity towards DPPH, ABTS and NO radicals and a wound healing assay was carried out to study the influence of the product on cell migration. The obtained results highlighted a significant barrier effect, with a reduction in caffeine penetration equal to 65.3%, the ability to both repair and prevent the damage caused by an acid insult, confirmed by a good transepithelial resistance for the tissue treated with the tested item, and the capacity to promote wound healing. Furthermore, the tested product showed good antioxidant and anti-inflammatory properties in the performed radical scavenging assays. These findings support the use of REF-FTP78 in the treatment of GERD.

## Introduction

Gastroesophageal reflux disease (GERD) represents a widespread clinical problem involving the abnormal ascent of gastric contents into the esophagus or beyond, into the oral cavity including larynx, and it is globally defined as a “condition which develops when the reflux of stomach contents causes troublesome symptoms and/or complications”^1^. A significant percentage equal to 13.98% of the global population suffers from this common condition with relevant variations between countries and a prevalence as high as 10–20% in the western world^2^. GERD can be classified into two main categories, such as erosive reflux disease (ERD) and non-erosive reflux disease (NERD), whose pathogenesis has not been thoroughly investigated^3,4^. This classification is based on the presence or not of esophageal injury observed using endoscopy as diagnostic technique.

GERD pathophysiology is characterized by a multifactorial nature and it is associated with an imbalance between the refluxate noxious effects and an ineffective defense system, which can be accompanied or not to a faulty valvular mechanism at the level of the esophagogastric junction^5^.

The most common GERD symptom is a retrosternal burning sensation called heartburn. An estimated 20–40% of patients complaining of heartburn, indeed, will receive a diagnosis of GERD^6^. This symptom is associated with prostaglandin E_2_ (PGE_2_) production in GERD patients, but the productive mechanism is unclear. A recently published study highlighted that PGE_2_ production is increased following exposure to weak acids via TRPV_4_/ERK/cPLA_2_ in oesophageal epithelial cells suggesting a role in GERD symptoms such as heartburn^7^. Other characteristic manifestations of this condition include regurgitation, chest pain, sore throat, cough, globus sensation and dysphagia.

The repeated exposure of esophagus to the gastric contents, which mainly consist of hydrochloric acid and pepsin, can lead to a tissue damage. Hydrochloric acid, indeed, causes an alteration of the junctions between the esophageal epithelial cells leading to dilated intercellular spaces and increased paracellular permeability^8^. The result is a diffusion into the intercellular space firstly of hydrogen ions causing acidification together with cell necrosis, and secondly of chloride ions followed by the development of an osmotic imbalance. In addition, the acidification of the intercellular space stimulates pH-sensitive nociceptors resulting in a pain sensation^9^.

Most patients require continuous and long-term treatment for the management of GERD to counteract the symptoms onset and/or promote esophageal healing. Despite different drugs, including proton pump inhibitors and H_2_ receptor blockers, available on the market as first-line treatment, relevant side effects have been observed resulting in an increase in the demand for alternative approaches^10,11^. A recent study reports on prolonged and multifaceted changes in immune cells following the use of H_2_ receptors blockers, such as ranitidine, which should contribute to a modified immune response^12^.

Moreover, the removal from the market of all formulations containing the histamine H_2_-receptor antagonist ranitidine, which was widely used in the treatment of GERD and other conditions involving acid reflux into the esophagus, has been contributed to direct the research towards the development of new products. Ranitidine-containing medicinal products, indeed, has been withdrawn in April 2020 after Food and Drug Administration (FDA) request due to the presence above levels considered acceptable of an impurity called N-nitrosodimethylamine (NDMA) classified as a probable human carcinogen^13^.

In this context, medical devices based on natural extracts are attracting significant interest.

REF-FTP78 (Manufacturer Labomar S.p.A.) is an innovative class IIb medical device for the treatment of GERD and commercially available with different trade names. The main components are polysaccharides from *Aloe Barbadensis* Leaf Juice and fucoidans from edible brown seaweeds such as *Undaria pinnatifida* and *Fucus vesiculosus*. The formula is completed by the presence of hydroxypropyl methylcellulose (HPMC) and xanthan gum (XG), which improve the mucoadhesive properties, and potassium citrate and magnesium carbonate that act as antacids.

The wound healing properties of *Aloe vera* are well known^14,15^. Several studies, indeed, reported on the ability of *Aloe vera* gel to promote wound healing increasing collagen and glycosaminoglycan synthesis^16–19^. Acemannan represents the major polysaccharide of *Aloe vera* gel^20^ and plays a key role in wound healing process inducing fibroblast proliferation and collagen expression^21^. In addition, *Aloe vera* has showed gastroprotective properties, due to the inhibition of gastric acid secretion^22^, reduction of oxidative stress and inflammation and improvement of gastric histopathology^23–25^, resulting effective in reducing GERD symptoms with no adverse events^26^.

Fucoidans are sulfated polysaccharides isolated primarily from brown seaweeds and mainly composed of fucose and sulfate groups, but also other compounds are present in their structure such as uronic acid and monosaccharides including glucose, mannose and rhamnose. Their biological properties are strictly dependent on molecular weight, structure, monosaccharide composition and content and location of sulphate groups^27^. As widely reported in literature, these polysaccharides, indeed, are characterized by several interesting biological properties including anti-inflammatory, antioxidant and wound healing activities^28–33^, which all contribute to the management of GERD, exerting a protective effect from oxidative stress and inflammation implicated in its pathophysiology, and mucosal damage caused by gastric refluxate.

These properties together with the mucoadhesive and soothing properties of polysaccharides from *Aloe vera* and brown seaweeds, strengthened by the film-forming behaviour of hydroxypropyl methylcellulose^34,35^, allow to improve the protective action of the studied medical device through a “barrier effect”.

The aim of the present study was to assess the efficacy of REF-FTP78 in the management of GERD devoting a particular attention to the mucoadhesive and, thus, the barrier effect combined with wound healing, antioxidant and anti-inflammatory activities.

## Results and discussion

### Evaluation of the film-forming properties and barrier effect on in vitro reconstructed human esophageal epithelium

The performed assay aims to investigate the film-forming and, thus, the barrier effect exerted by the medical device under examination. For this purpose, an in vitro reconstructed human esophageal epithelium was treated with REF-FTP78 for 3 h and a multimodal approach was used to evaluate both the barrier integrity before (t_0_) and after (t_1_) the treatment by TEER measurement and tissue permeability by caffeine penetration and Lucifer Yellow assay.

**Figure 1:**
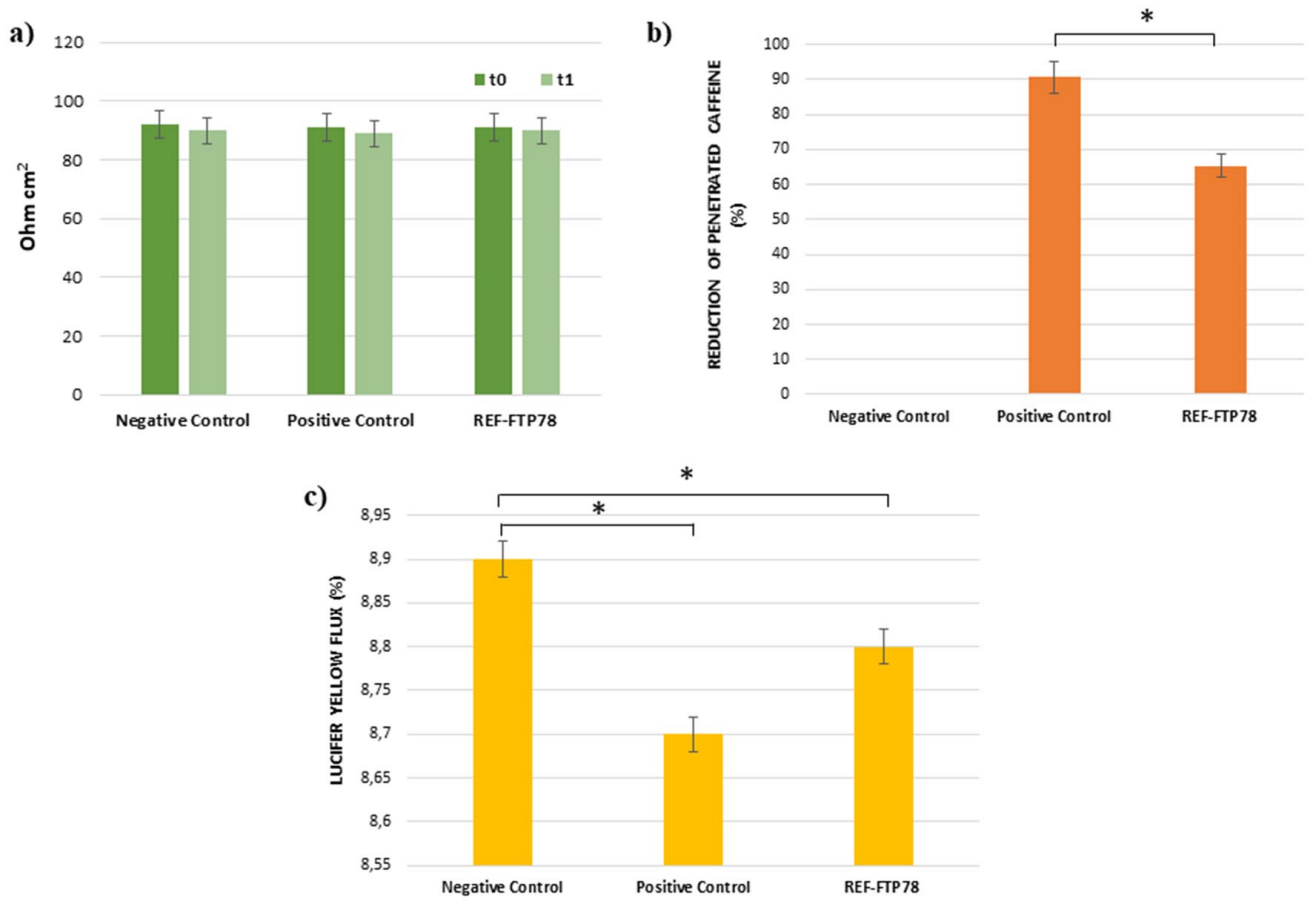
**(a)** TEER; **(b)** reduction of caffeine penetration expressed as percentage; **(c)** Lucifer Yellow flux. *P < 0.05.

TEER results, expressed as Ohm cm^2^, are shown in Fig. 1a and confirmed that the product does not affect the paracellular flow and, thus, the barrier integrity. TEER values recorded after the treatment with REF-FTP78 at the end of the caffeine permeation study (t_1_ = 6 h), indeed, do not differ from those measured at t_0_. This behavior was also observed for the negative and positive controls.

Caffeine is widely employed as model molecule in in vitro percutaneous absorption studies devoted to the evaluation of tissue permeability due to its capability to cross also the intact epithelial barrier. Therefore, this compound is very useful to determine the film-forming behavior of a product. Based on these considerations, the caffeine permeation of reconstructed human esophageal epithelium treated with REF-FTP78 was assessed and compared to a negative control consisting of saline solution. The detected reduction in caffeine penetration (%) was reported in Fig. 1b and, as it is possible to observe, the tested item allows to reduce the penetration of this molecule by 65.3 ± 0.3% confirming its film-forming properties and a persistent barrier effect, which is retained after 3 h from the treatment. Vaseline, which was used as positive control due to its occlusive properties, was able to inhibit the caffeine diffusion by 90.6 ± 0.5%.

The Lucifer Yellow (LY) assay is employed to evaluate the integrity of cell junctions. LY, indeed, is a fluorescent dye characterized by a very low cell permeability but, when junctions are injured, LY paracellular flux increases. In the present study, LY paracellular flux was measured after the treatment with REF-FTP78 at the end of the caffeine permeation study. As shown in Fig. 1c, the obtained results for the tested product and negative and positive controls are comparable supporting that the integrity of the epithelium barrier was preserved also after the exposure to the medical device, which did not cause direct damage to the tissue.

### Ex vivo evaluation of the barrier effect on esophageal mucosal damage and mucoadhesive properties

In literature is widely reported that the esophageal mucosal damage induced by the perfusion of acidic solutions in the presence or absence of pepsin represents a model of GERD lesions^36^. In the present study, this model, which is considered suitable for ex vivo studies aimed to evaluate the adhesive behavior of therapeutics^37^, was adopted to investigate the barrier effect and the mucoadhesive properties of REF-FTP78 using different experimental conditions. For this purpose, indeed, hydrochloric acid solutions in the presence or not of pepsin were perfused for 30 and 60 min to induce the esophageal mucosal damage.

The esophageal mucosal permeability was evaluated by using Evans blue, which is a high molecular weight dye already employed to investigate mucosal permeability of jejunum and colon in rat and mouse, respectively^38,39^.

**Figure 2:**
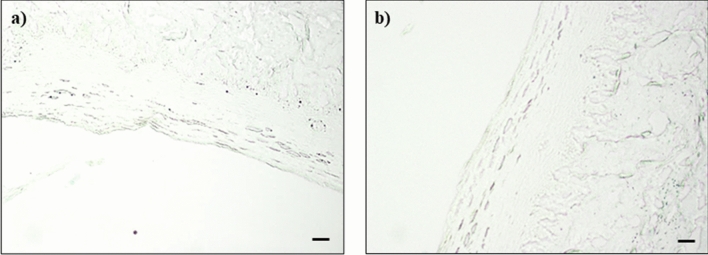
Negative control: undamaged mucosa after 30 **(a)** and 60 **(b)** minutes perfusion with saline solution.

**Figure 3:**
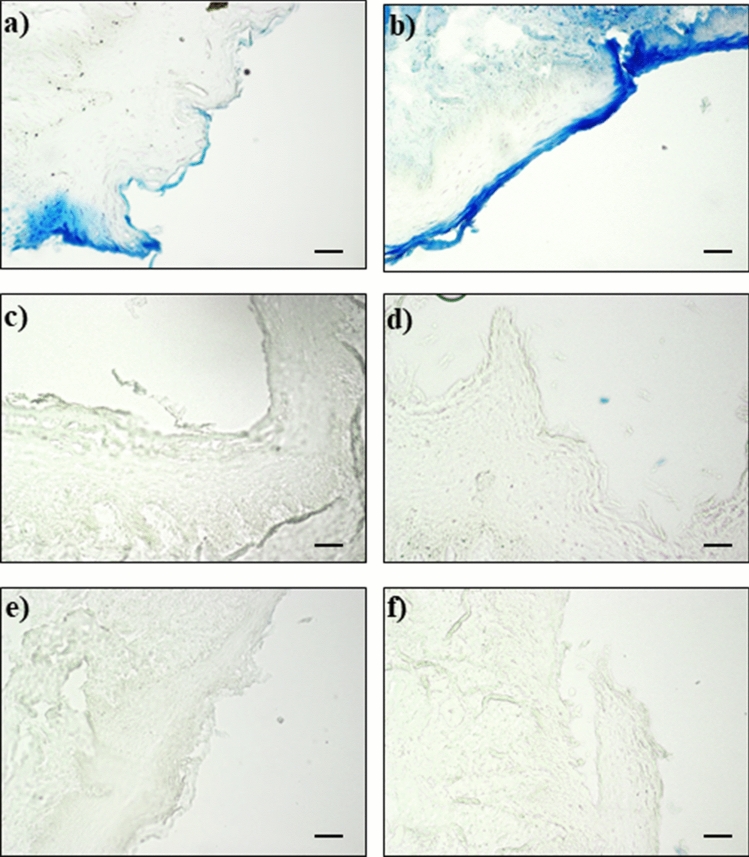
Damaged mucosa after 30 **(a)** and 60 **(b)** minutes perfusion with acid solution, 30 **(c)** and 60 **(d)** minutes perfusion with acid solution followed by 10 min perfusion with REF-FTP78 and 30 **(e)** and 60 **(f)** minutes perfusion with acid solution followed by 10 min perfusion with REF-FTP78 and a washing step of 30 s with saline before EB staining. Scale bar: 50 µm.

**Figure 4:**
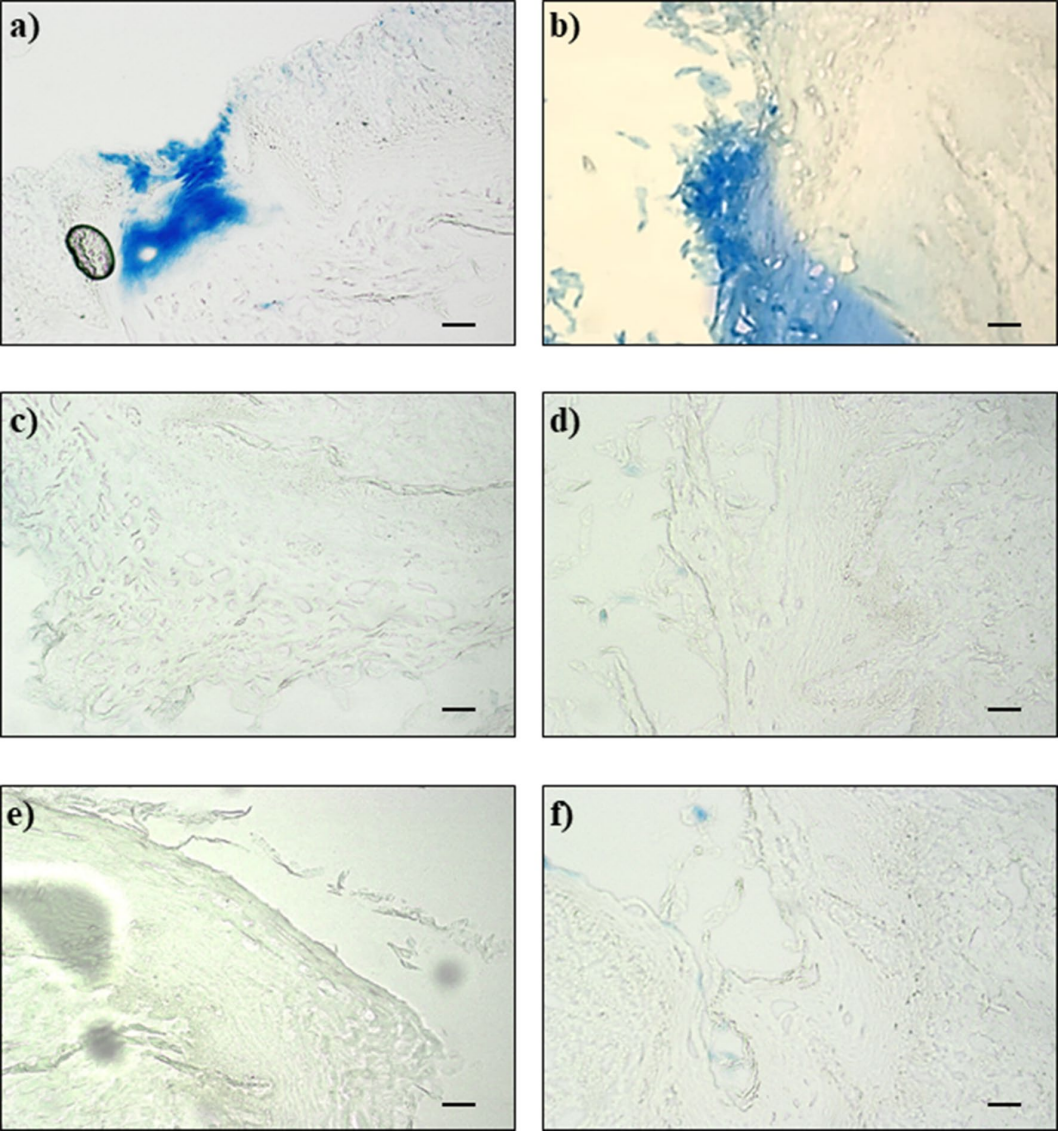
Damaged mucosa after 30 **(a)** and 60 **(b)** minutes perfusion with acid solution in the presence of pepsin, 30 **(c)** and 60 **(d)** minutes perfusion with acid solution in the presence of pepsin followed by 10 min perfusion with REF-FTP78 and 30 **(e)** and 60 **(f)** minutes perfusion with acid solution in the presence of pepsin followed by 10 min perfusion with REF-FTP78 and a washing step of 30 s with saline before EB staining. Scale bar: 50 µm.

The results of EB staining were reported in the following Figs. 2, 3 and 4.

No EB staining was observed for the undamaged mucosa acting as negative control sample (Fig. 2a,b); on the contrary, the EB staining was evident in the case of injured mucosa (Figs. 3a,b, 4a,b).

Scale bar: 50 µm.

In particular, the induced histological damage and, thus, the severity of the observed lesions depend on the adopted perfusion time. The histological damage was evident after a 30 min perfusion with the hydrochloric acid solution (Fig. 3a), while an increased and progressive damage involving the inner mucosal layers was observed after 60 min (Fig. 3b).

The perfusion with the acid solution containing pepsin induced a significant histological damage as early as 30 min (Fig. 4a) and a severe histological lesion was observed after 60 min (Fig. 4b).

Perfusion with REF-FTP78 after damaging with the acid solution in the presence or not of pepsin was able to completely prevent the EB staining in all the studied mucosa samples (Figs. 3c,d, 4c,d) confirming the ability of the medical device to form a barrier on the damaged mucosa and, thus, avoid the EB diffusion and penetration.

Moreover, the observed effect was retained also after a washing step of 30 s with saline before the perfusion of EB solution attesting the mucoadhesive properties of the product, which allow to obtain a lasting barrier effect accompanied by the prevention of the injured mucosa permeability (Figs. 3e,f, 4e,f). The increased permeability due to the damage induced by the gastroesophageal reflux, indeed, represents one of the key mechanisms responsible for the onset of GERD symptoms such as heartburn.

### Evaluation of regenerative and protective properties on in vitro reconstructed human esophageal epithelium

#### Morphological studies

The regenerative and protective properties of REF-FTP78 were also tested on an in vitro reconstructed human esophageal epithelium, which represents a biological model reproducing the human esophageal epithelium morphology.

**Figure 5:**
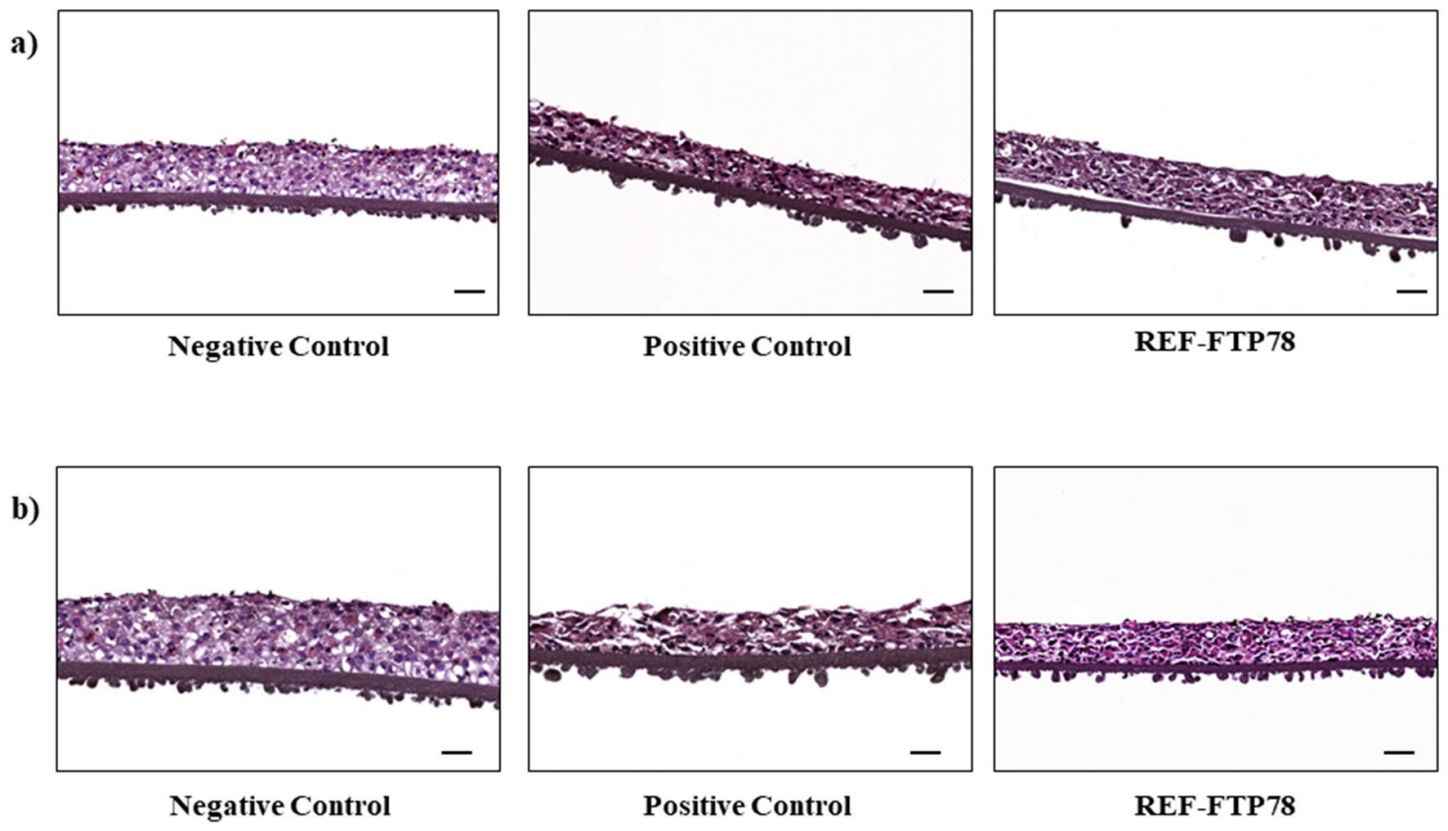
Morphological evaluation of the tissue inserts treated: (**a)** with HCl solution for 1 min followed by REF-FTP78 for 24 h; (**b)** with REF-FTP78 for 1 h followed by HCl solution for 10 min. Scale bar: 50 µm.

In order to investigate the regenerative ability of the tested product, the tissue inserts were treated with the hydrochloric acid solution for 1 min, washed with PBS and, then, treated with REF-FTP78 for 24 h. The histological analysis (Fig. 5a) revealed a good regenerative capacity exerted by the medical device, compared to the positive control, in terms of reduction of cellular degeneration, epithelial erosion as well as necrosis after 1 min acid insult (Table 1).

**Table 1:** Morphological evaluation of the treated tissue inserts.

Sample	Cellular degeneration	Erosion	Necrosis
Negative control 1 min HCl	−	−	−
Positive control 1 min HCl	**++**	**++**	**++**
REF-FTP78 1 min HCl	**+**	**+**	**+**
Negative control 10 min HCl	−	−	−
Positive control 10 min HCl	**+++**	**+++**	**++**
REF-FTP78 10 min HCl	**+**	**+**	−

−: absent (0%); +: mild (< 10%);++: moderate (≥ 10 to < 40%); +++: serious (≥ 40%).

The percentage indicates the cell counts of three different sections of three different experiments performed by two independent operators.

In a parallel experiment, the inserts were previously treated for 1 h with REF-FTP78 and, then, exposed to the HCl solution for 10 min. In this case, the histological analysis showed a higher preservation of the tissue parameters for the REF-FTP78 treated samples, compared to the positive control, after 10 min HCl exposition (Fig. 5b, Table 1).

The performed morphological studies confirmed the ability of the product under examination to both repair and prevent the damage caused by an acid insult and to interact with the esophageal epithelium forming a protective barrier.

#### Evaluation of the barrier effect and tissue permeability following damage induced by hydrochloric acid

In the present study, the film-forming properties and the barrier effect of REF-FTP78 were explored under different experimental conditions. The experimental design reported in “Evaluation of the film-forming properties and barrier effect on in vitro reconstructed human esophageal epithelium” section involved the treatment of an in vitro reconstructed human esophageal epithelium with the medical device for 3 h, which confirmed that the product does not affect the integrity of the epithelium barrier (Fig. 1a,c) and, at the same time, it was able to reduce caffeine penetration due to its film-forming properties and a persistent barrier effect (Fig. 1b).

In this section, film-forming and barrier properties of REF-FTP78 were further investigated following damage induced by hydrochloric acid using both TEER measurement and the Lucifer Yellow assay.

To this aim, an in vitro reconstructed human esophageal epithelium was firstly treated with the product under examination for 1 h and, then, with an HCl solution for 10 min.

**Figure 6:**
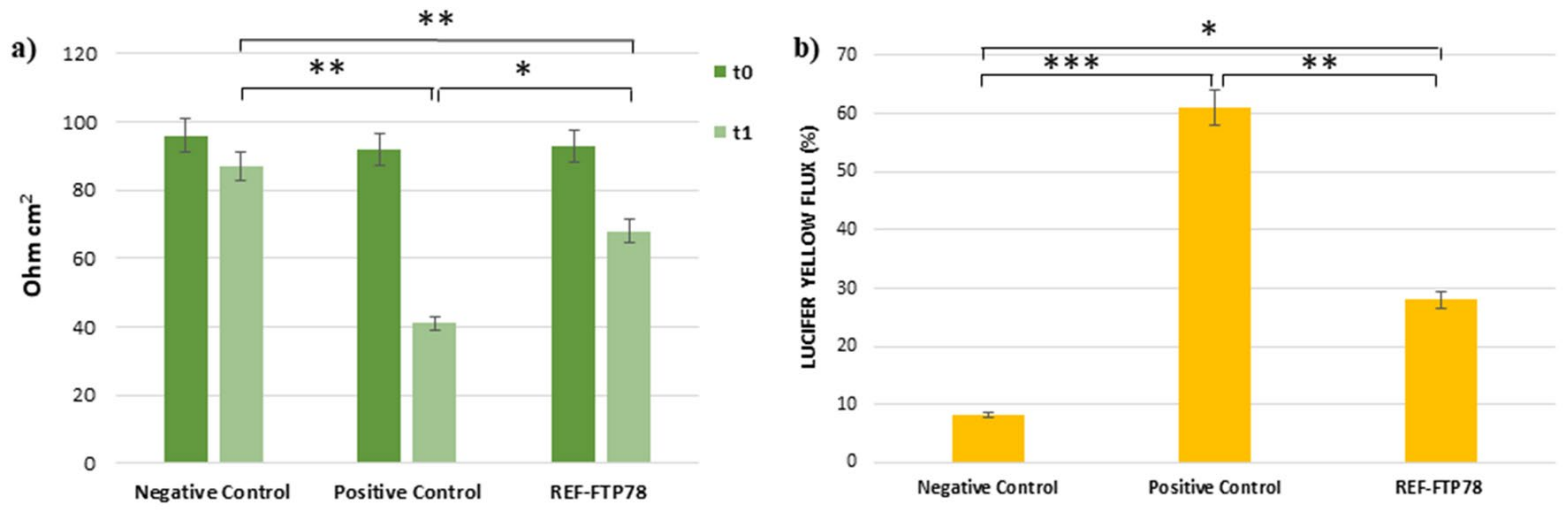
**(a)** TEER; **(b)** Lucifer Yellow flux. *P < 0.05; **P < 0.005; ***P < 0.001.

TEER allowed to evaluate the barrier integrity through the ion paracellular flux measurement before (t_0_) and after (t_1_) the treatment. The obtained results, reported in Fig. 6a, highlighted a good transepithelial resistance for the sample treated with the tested item, which helps to preserve the epithelial mucosa integrity compared to the positive control.

After the treatment with REF-FTP78, Lucifer Yellow flux was also measured (Fig. 6b) and a good reduction in the passage of LY was observed compared to the positive control. This result is indicative of the ability of the tested medical device to counteract the damage due to the acidic insult and, thus, maintain the barrier functionality.

These findings support the biocompatibility of the tested medical device, which does not damage the tissue as observed in Fig. 1, and its efficacy in preserving the tissue from the noxious components of refluxate hindering the damage induced by the acidic insult (Fig. 6).

### Assessment of the wound healing effect

Wound healing is a complex and dynamic process consisting of several cellular events aimed at repairing and regenerating the damaged skin site. The first phase of this process involves the formation of a clot, which is accompanied by other events such as vascularization, introduction of inflammatory immune cells and cell proliferation, migration and differentiation to close the wound^40^.

In this study, the ability of REF-FTP78 to promote the regenerative process was explored by the in vitro wound healing assay. For this purpose, after the formation of a wound field with a defined gap, cells were treated with the tested product and monitored for migration and proliferation into the wound field. Percent of wound closure and migration rate were calculated according to the following Eqs. (1) and (2), respectively:

1 $$Percent \,of\, wound \,closure \left(\%\right)= \frac{Migrated\, cell\, surface\, area}{Total \,surface\, area} \times 100,$$

where migrated cell surface area is the length of cell migration (mm) × 2 × length and total surface area is equal to 0.9 mm × length;

2 $$Migration \,rate= \frac{Length \,of\, cell \,migration\, (\text{nm})}{Migration \,time\, (\text{h})}.$$

**Figure 7:**
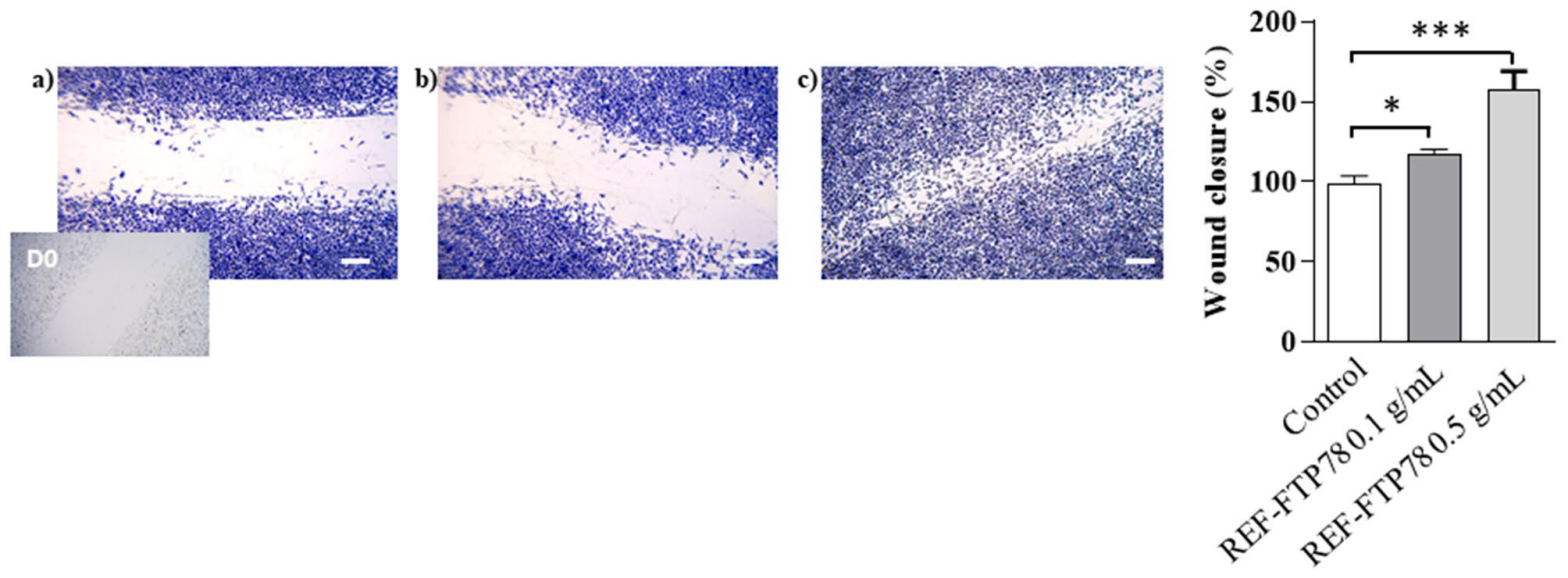
In vitro wound healing assay: **(a)** control sample (untreated cells); **(b)** REF-FTP78 0.1 g/mL and **(c)** REF-FTP78 0.5 g/mL. The percentage of wound closure has been represented on histograms calculated using ImageJ software version 1, 51 Square, time 0. *P < 0.05; **P < 0.005; ***P < 0.001. Scale bars: 25 µm.

In the adopted experimental conditions, REF-FTP78 determined after 48 h of treatment a Percent of Wound Closure equal to 27.3% and 61.0%, and a Migration Rate of 2.9 and 6.3 nm/h at a concentration of 0.1 and 0.5 g/mL, respectively. As it is possible to observe in Fig. 7, indeed, the wound healing in the scratched monolayer was faster and favored when cells were treated with REF-FTP78 compared to untreated cells. Therefore, the studied medical device exerts a significant wound healing effect at both the tested concentrations.

### Evaluation of the antioxidant and anti-inflammatory activities

Different assays are required to investigate the antioxidant capacity of a product against multiple types of free radicals such as lipophilic and hydrophilic species.

The DPPH assay is a simple, accurate and low-cost method developed by Blois in 1958 for the evaluation of the antioxidant properties of a sample^41^. This method is based on the measurement of the scavenging activity of the tested item towards DPPH, which is a stable lipophilic free radical that does not need to be generated^42–44^. The delocalisation of the spare electron over the radical molecule, indeed, contributes to the stabilization of the radical itself and provides a deep violet colour with an absorption maximum band at around 517 nm. On mixing the radical with an antioxidant sample able to donate a hydrogen atom, the electron on DPPH nitrogen atom pairs off leading to the reduced corresponding hydrazine and to a loss of colour. Therefore, the scavenging activity is measured as the decrease in DPPH absorbance.

The DPPH inhibition percentage was calculated according to Eq. (3):

3 $$Inhibition\, \%= \frac{{A}_{0}-{A}_{1}}{{A}_{0}}\times 100,$$

where A_0_ is the absorbance of a control prepared in the same conditions, but without any sample, and A_1_ is the absorbance of the tested item. Then, the obtained results were used to calculate the IC_50_, which is the sample concentration required to inhibit 50% of radical. This value was equal to 139.38 ± 0.78 mg/mL confirming the good antioxidant properties of REF-FTP78 in terms of scavenging activity towards a lipophilic radical.

In this study, ABTS assay was also carried out in order to evaluate the antioxidant properties of the medical device under examination towards hydrophilic radicals^45^.

ABTS^·+^ radical cation is generated by mixing potassium persulfate and ABTS and leaving the resulting solution overnight. The interaction between the so pre-generated radical and the antioxidant sample involves an electron transfer mechanism leading to the ABTS^·+^ reduction by the antioxidant, which can be detected by a discoloration. The scavenging activity was measured as the decrease in ABTS absorbance and Eq. (3) was used to calculate the ABTS inhibition percentage resulting in an IC_50_ of 16.49 ± 0.73 mg/mL.

The observed good antioxidant properties of the studied medical device are ascribable to its main components such as *Aloe Barbadensis* Leaf Juice and fucoidans from *Undaria pinnatifida* and *Fucus vesiculosus* extracts. The antioxidant properties of *Aloe vera*, indeed, are well known and the significant antioxidant activity of aloe extracts in terms of DPPH scavenging is widely reported in literature^46^ as well as the presence of a potent antioxidant compound characterized by an antioxidant activity similar to that of α-tocopherol as assessed in vitro using rat brain homogenates^47^. In addition, the sulfate content of fucoidans from *Undaria pinnatifida* and *Fucus vesiculosus* extracts, which are highly polydisperse sulfated polysaccharides, affects their biological properties including the antioxidant activity.

Taking into account that the lower the IC_50_, the higher the antioxidant properties of the tested sample, the scavenging activity of REF-FTP78 was more pronounced against hydrophilic radicals, such as ABTS, than lipophilic ones.

REF-FTP78 anti-inflammatory properties were also investigated by measuring the nitric oxide (NO) radical scavenging capacity in sodium nitroprusside/Griess reagent system^48^.

Nitric oxide is a free radical exerting a key role in the regulation of cell functions involved in immune and inflammatory responses^49^. Under physiological conditions NO levels are low; on the contrary, during inflammatory processes NO production increases resulting in a potential tissue damage due to the activation of catabolic events including expression of proinflammatory cytokines, such as interleukin 6 (IL-6), and prostaglandins (PGs), which represent pivotal factors in inflammatory processes. Therefore, samples able to inhibit NO radicals are characterized by anti-inflammatory properties.

In the performed assay, NO radicals generated from SNP interacted with oxygen leading to the production of nitrite ions, which were assessed by mixing with Griess reagent. The absorbance of the chromophore produced during the diazotization of nitrite ions with sulfanilamide and subsequent coupling with naphthylethylenediamine dihydrochloride was measured at 546 nm.

The obtained results showed the ability of REF-FTP78 to inhibit NO radicals in a concentration-dependent manner with an IC_50_ of 245.45 ± 0.67 mg/mL.

## Conclusion

The present study aimed to assess the efficacy of REF-FTP78, a class IIb medical device based on polysaccharides from *Aloe Barbadensis* and fucoidans from brown seaweeds, for the management of symptoms associated with gastroesophageal reflux.

This medical device acts throughout a multimodal mechanism of action involving the formation of a mucoadhesive polymeric film on the esophageal mucosa, which represents a mechanical barrier, and regenerative, antioxidant and anti-inflammatory abilities due to the presence of polysaccharides from *Aloe Barbadensis* and fucoidans from *Undaria pinnatifida* and *Fucus vesiculosus*. In particular, the product acts by contact with the esophageal mucosa carrying out a mechanical action due to the formation of a physical barrier consisting of a protective film, which is retained over time.

Overall, REF-FTP78 represents an effective alternative, based on natural extracts and bioadhesive polymers, to traditional drugs in the treatment of gastroesophageal reflux able to exert its function through a mechanical action that does not affect the physiological balance of the esophageal epithelium.

## Methods

### Materials

2,2-diphenyl-1-picrylhydrazyl radical (DPPH), 2,2′-azinobis-(3-ethylbenzothiazoline-6-sulfonic acid) (ABTS), potassium persulfate, sodium nitroprusside (SNP), Griess reagent, disodium hydrogen phosphate, sodium dihydrogen phosphate, sodium chloride, sodium phosphate, Vaseline, caffeine, hydrochloric acid, pepsin from porcine gastric mucosa, Evans blue (EB), fetal bovine serum (FBS), formaldehyde, hematoxylin, eosin, Lucifer Yellow (LY), Dulbecco’s modification of eagle’s medium (DMEM) and penicillin–streptomycin were obtained by Sigma-Aldrich s.r.l. (Milan, Italy).

All solvents were reagent or HPLC grade and obtained from VWR (Milan, Italy).

REF-FTP78 was supplied by Labomar S.p.A. (Istrana, TV, Italy) as 10 mL stick pack.

CytoSelect™ 24-Well Wound Healing Assay Kit (Catalog Number CBA-120) was provided by Cell Biolabs, Inc. (San Diego, CA, USA).

Swine esophagi from commercially available pigs slaughtered for food purposes were supplied by a local slaughter house (Cosenza, CS, Italy) replacing the slaughter of animals for tissue sampling according to the 3Rs (Reduction, Replacement and Refinement). Therefore, the ethical approval was not needed as no animal had been sacrificed for experimental purposes.

Reconstructed human esophageal epithelium (HO2E/S/5, batch N° 21 HO2E 001) and the maintenance medium (21 SMM 018) were supplied by EpiSkin (Lyon Cedex, France).

### Cell culture

Balb/c 3T3 clone A31 (ATCC^®^; CCL163™) mouse fibroblasts were obtained from the American Type Culture Collection (ATCC, Manassas, VA, USA). Cells were cultured in DMEM supplemented with 10% Calf Bovine Serum (CBS; ATCC, Manassas, VA, USA) and 1% penicillin–streptomycin (10,000 unit/mL) at 37 °C in a 5% CO_2_ atmosphere.

### Evaluation of the film-forming properties and barrier effect on in vitro reconstructed human esophageal epithelium

#### Trans epithelial electrical resistance (TEER) measurement and permeability studies

The present assay wants to investigate the film-forming and, thus, the barrier effect of REF-FTP78 on an in vitro reconstructed human esophageal epithelium.

The employed human esophageal epithelium was in vitro reconstructed after 5 days of airlift culture of K510 cell line on inert polycarbonate filters. On day 5, the inserts containing the tissues were placed at room temperature in a multi-well plate filled with an agarose nutrient solution. After that, tissues were removed from the agarose solution using a sterile airflow cabin and quickly transferred to 24-well plates previously filled with the maintenance medium (1 mL/well) at room temperature and incubated at 37 °C, 5% CO_2_ and saturated humidity.

The Trans Epithelial Electrical Resistance (TEER) of each tissue was measured using a Millicell-ERS-2 instrument (Millipore, Massachusetts, United States, range 0–20 kΩ) before (t_0_) the treatment with the medical device to confirm the integrity of the barrier membrane^50,51^. Then, the reconstructed human esophageal epithelium was treated with 30 μL of the tested product at room temperature for 3 h, while NaCl 0.9% (w/v) and Vaseline were used as negative and positive controls, respectively.

For the permeability study, caffeine was used as reference compound according to the OECD test guideline 428^50,52^.

After the treatment, tissues were transferred in a 12-well plate previously filled with 1 mL/well of saline solution (receptor compartment). Then, 0.1 mL of a caffeine solution in water (1% w/v) were introduced into the donor compartment and applied on the treated Esophageal Epithelium without washing out the tested item. Tissues were incubated at 37 °C and 5% CO_2_ and, after 3 h, the receptor fluid was withdrawn and samples were analyzed by HPLC for caffeine determination as reported below.

The results were expressed as reduction of penetrated caffeine (%) compared to the negative control.

At the end of the experiment (6 h), the excess of sample was removed and TEER was measured (t_1_).

#### HPLC analysis

HPLC analysis was performed using a Varian 900-LC (Varian Inc., CA, United States) equipped with an autosampler, a quaternary pump and an UV detector set at 280 nm. A 250 × 4.6 mm C-18 VivaTM column, particle size 5 µm (Restek, Barcelona, Spain) was employed. The flow rate was 1.0 mL/min and injection volume was 30 μL. The mobile phase was an 80:20 (v/v) mixture of HPLC-grade water containing sodium phosphate (0.05 M) and methanol. HPLC data were acquired using Galaxie™ Chromatography Software^53^.

#### Lucifer Yellow assay

After the caffeine permeation study, the removal of sample excess and TEER measurement, the permeability of treated tissues was also investigated according to the Lucifer Yellow assay^50,52^.

For this purpose, 0.5 mL of a 500 μM LY solution prepared in saline were added to the apical compartment, while 1.0 mL of saline solution was placed into the basolateral compartment. After incubation at 37 °C for 1 h, the passage of the fluorescent dye from the apical to the basolateral compartment was determined by spectrofluorimetric analysis (excitation at 428 nm and emission at 535 nm) using a Synergy H1 spectrofluorometer (Hybrid Reader, BioTek Instruments, Agilent, CA, United States). The flux was calculated according to the following Eq. (4):

4 $$LY\, Flux\, \%=\left({RFU}_{BL}/{RFU}_{AP t=0}\right) \times 100,$$

where RFU_BL_ is the measurement of fluorescence in the basolateral compartment and RFU_AP t=0_ is the mean of the LY solution RFU.

### Ex vivo evaluation of the barrier effect on esophageal mucosal damage and mucoadhesive properties

Swine esophagi were supplied by a local slaughter house and transferred within 2 h upon slaughter in cooled saline to the research laboratory. Each esophagus was washed with water and, then, the mucosa was isolated and kept in vital condition on an oblique polystyrene surface with an inclination of about 45 degrees and at 37 °C in a thermostated hood.

The top of the esophageal tube was tied with a surgery suture to a syringe attached to a perfusion pump, while the bottom was closed for 5 min with a surgical knot allowing to fill the tube with the damaging solution.

The assay was performed as reported in literature inducing the esophageal mucosal damage perfusing esophagi at 37 °C for 30 and 60 min with hydrochloric acid solutions (0.1 N) in the presence or not of pepsin (2000 U/mL) and setting the perfusion rate at 1 mL/min^36,54^. Then, the Evans blue (EB) dye was used to evaluate the permeability of both undamaged (negative control) and damaged esophageal mucosa. For this purpose, esophagi were perfused with a 10 mg/mL EB solution in sterile saline for 10 min at a flow rate of 1 mL/min.

The damaged mucosa was previously washed with saline for 5 min and divided into three portions. The first one was used as positive control and perfused with EB; the second one was perfused for 10 min with REF-FTP78 (1 mL/min) followed by EB and the last one was perfused for 10 min with REF-FTP78 (1 mL/min), then washed with saline for 30 s, and finally perfused with EB in the aim to evaluate the mucoadhesive properties of the tested product. Light microscopy was used to evaluate the EB staining.

### Evaluation of regenerative and protective properties on in vitro reconstructed human esophageal epithelium

#### Morphological studies

These studies aim to investigate the regenerative and protective properties of the tested medical device on an in vitro reconstructed human esophageal epithelium following damage induced by a hydrochloric acid solution (10% v/v, pH 1.1). For this purpose, two different experimental protocols were adopted.

The first one involved the tissues treatment with a hydrochloric acid solution (10% v/v, pH 1.1) for 1 min. Then, the tissues were washed with PBS (phosphate buffered saline) and, finally, treated with REF-FTP78 for 24 h in the aim to evaluate the regenerative properties of the tested product. Tissue inserts treated with NaCl 0.9% (w/v) or the HCl solution for 1 min, washed with PBS and treated with physiological saline for further 24 h served as negative and positive controls, respectively.

The second experimental procedure was carried out to evaluate the protecting effect of the medical device by treating the inserts for 1 h with the product under examination and, then, with the HCl solution for 10 min. Tissue inserts treated for 1 h with NaCl 0.9% and then, with saline or HCl solution for further 10 min were used as negative and positive controls, respectively.

After each treatment, the tissues were fixed in a 4% formaldehyde solution for histological analysis and morphological studies were performed on the in vitro reconstructed human esophageal epithelium by hematoxylin–eosin staining.

#### Evaluation of the barrier effect and tissue permeability following damage induced by hydrochloric acid

This assay aims to evaluate the barrier effect of the tested item on an in vitro reconstructed human esophageal epithelium following damage induced by a hydrochloric acid solution (10% v/v, pH 1.1). The film-forming properties of REF-FTP78 were investigated by a multi-parameter approach including both TEER measurement and tissue permeability assessment by Lucifer Yellow assay.

For this purpose, the tested product was applied on the surface of the epithelium for 1 h before the treatment for 10 min with the acid solution. Tissue inserts treated with physiological saline for 1 h and, then, with saline or HCl solution for 10 min served as negative and positive controls, respectively.

The TEER of each tissue was measured before the treatment with the medical device (t_0_), in the aim to confirm the integrity of the barrier membrane, and at the end of the experiment (t_1_).

After TEER measurement, the excess sample was removed from the tissue surface by washing and, then, the permeability was investigated according to the Lucifer Yellow assay as reported before.

### Assessment of the wound healing effect

The in vitro evaluation of the wound healing effect of REF-FTP78 was investigated using the CytoSelect™ 24-Well Wound Healing Assay Kit according to the manufacturer’s instructions^55^.

A fibroblasts Balb/c 3T3 suspension containing 1.0 × 10^6^ cells/mL in medium containing 10% (v/v) FBS was prepared. Then, 500 μL of the cell suspension were added to each well with the insert in place and cells were incubated at 37 °C in a 5% CO_2_ atmosphere until a monolayer was formed. The insert was gently removed to generate a wound field with a defined gap of 0.9 mm between the cells. Then, the medium was slowly aspirated from the wells, discarded and the wells were washed with fresh medium in order to remove dead cells and debris. After the washing step was complete, cells were treated with the tested product and incubated at 37 °C in a 5% CO_2_ atmosphere. The wound closure was monitored by cell staining using an Olympus BX41 microscope (Olympus, Tokyo, Japan), and the images were taken with CSV1.14 software using a CAM XC-30 for image acquisition.

REF-FTP78 was tested at a concentration of 0.1 and 0.5 g/mL (dissolved in the medium) and compared to a control prepared under the same experimental conditions but without any sample.

### Evaluation of the antioxidant and anti-inflammatory activities

#### DPPH antioxidant assay

The antioxidant properties of REF-FTP78 in terms of scavenging activity toward the lipophilic radical 2,2-diphenyl-1-picrylhydrazyl radical (DPPH) were evaluated as previously reported in literature with slight modification^56^.

In a 10 mL volumetric flask, different amounts of the tested item (30–200 mg/mL) were introduced, mixed with 4 mL of a DPPH ethanol solution (188 μM) and diluted to the mark with ethanol. Samples were incubated in dark conditions for 15 min and, then, the absorbance was read at 517 nm after filtration using an Evolution 201 UV–Visible Spectrophotometer (Thermo Fisher Scientific, Waltham, MA, USA).

The assay was performed in triplicate and results expressed as mean ± SD.

#### ABTS antioxidant assay

The scavenging activity of REF-FTP78 towards the hydrophilic ABTS radical cation was also investigated.

The ABTS radical cation (ABTS^·+^) solution was prepared as previously reported in literature^48^.

In a 5 mL flask, different amounts of the tested product (5–40 mg/mL) were mixed with 4 mL of the ABTS solution and diluted to the mark with distilled water. The obtained samples were incubated in a water bath at 37 °C and protected from light for 5 min. Then, the absorbance was read at 734 nm after filtration using an Evolution 201 UV–Visible Spectrophotometer (Thermo Fisher Scientific, Waltham, MA, USA).

The experiment was carried out in triplicate and results expressed as mean ± SD.

#### Nitric oxide (NO) scavenging assay

Different amounts of REF-FTP78 (50–350 mg/mL) were introduced into 10 mL flasks and, then, 2.5 mL of a 5 mM sodium nitroprusside solution prepared in phosphate-buffered saline at pH 7.3 were added to each sample^48^. The reaction mixtures were diluted to 10 mL with PBS and incubated for 180 min in front of a visible polychromatic light source (25 W tungsten lamp) causing the spontaneous decomposition of SNP and, thus, the generation of nitric oxide. This last one interacted with oxygen to produce nitrite ions, which were measured by the Griess reaction. For this purpose, 0.5 mL of each incubation mixture were added to 0.5 mL of the Griess reagent and, after 5 min, the absorbance was measured at 546 nm using an Evolution 201 UV–Visible Spectrophotometer (Thermo Fisher Scientific, Waltham, MA, USA).

The sodium nitroprusside/Griess reagent assay was repeated in triplicate and results expressed as mean ± SD.

### Statistical analysis

All experiments were performed at least 3 times. Data were expressed as mean values ± SD and statistical significance between control and treated samples was analyzed using GraphPad Prism 8.3.0 (GraphPad Software, Inc.) software. Control and treated groups were compared using t-test or the one-way ANOVA with Tukey post hoc testing. Significance was defined as P < 0.05.
